# The Development of a Real-Time Recombinase-Aid Amplification Assay for Rapid Detection of African Swine Fever Virus

**DOI:** 10.3389/fmicb.2022.846770

**Published:** 2022-03-17

**Authors:** Yongshu Wu, Yang Yang, Yi Ru, Xiaodong Qin, Miaomiao Li, Zhixiong Zhang, Rui Zhang, Yijing Li, Zhidong Zhang, Yanmin Li

**Affiliations:** ^1^State Key Laboratory of Veterinary Etiological Biology, Lanzhou Veterinary Research Institute, Chinese Academy of Agricultural Sciences, Lanzhou, China; ^2^College of Veterinary Medicine, Northeast Agricultural University, Harbin, China; ^3^College of Animal Science and Veterinary Medicine, Southwest Minzu University, Chengdu, China

**Keywords:** African swine fever virus, real-time recombinase-aid amplification assay, nucleic acid detection, qPCR, portable instrument

## Abstract

African swine fever (ASF), caused by the African swine fever virus (ASFV), is an acute, deadly, infectious disease of domestic pigs and wild boars and has a tremendous negative socioeconomic impact on the swine industry. ASF is a notifiable disease to the World Organization for Animal Health (OIE). Currently, no effective vaccine or treatment against ASF is available. Early detection and rapid diagnosis are potentially significant to control ASF spread with the emerging ASFV mutant strains and non-classical symptoms. In this study, we developed a real-time recombinase-aid amplification (RAA) assay to detect the ASFV genome rapidly. Thirty samples were detected using commercial lysis buffer for DNA extraction and equipped with a portable testing instrument. The results showed that the sensitivity of RAA was 10^3^ copies per reaction at 95% probability in 9 min at 39°C. The method was universally specific for three strains of ASFV, and there was no cross-reaction with other pathogens, including foot-and-mouth disease virus (FMDV), classical swine fever virus (CSFV), porcine reproductive and respiratory syndrome virus (PRRSV), porcine circovirus 2 (PCV2), pseudorabies virus (PRV), and porcine parvovirus (PPV). The coefficient of variation (C.V) of repetitive experiments was 0%, and the coincidence rate was 100% compared to the real-time qPCR. 123 field samples were detected by the real-time RAA assay, and the results showed that the clinical coincidence rate of the real-time RAA assay was 98% compared to the real-time qPCR assay. The advantages of this method were as follows: the extraction of DNA can be performed on site, the DNA template is directly used, a small battery-powered instrument is easily available, and the on-site diagnostic process is finished within an hour. These suggest that this assay could be used to detect different genotypes of ASFV and play a vital role in the control of ASF.

## Introduction

African swine fever (ASF) is caused by the African swine fever virus (ASFV) and is an acute, contagious, and hemorrhagic infectious disease, with a mortality rate reaching up to 100% (Galindo and Alonso, 2017). ASFV is a double-stranded DNA virus with a complex molecular structure and is the only member of the Asfarviridae family (Galindo and Alonso, 2017). ASF is a notifiable disease to the World Organization for Animal Health (OIE) due to its serious impact on pig production and related industries. The clinical symptoms include high fever, depression, anorexia, cyanosis, vomiting, nose bleeding, and bloody diarrhea, similar to classical swine fever (CSF) and porcine reproductive and respiratory syndrome (PRRS) and other swine diseases, which indicate that laboratory diagnosis is required (Blome et al., 2013). Twenty-four genotypes were presented based on sequence variation in the C-terminal region of the *B646L* gene that mainly encodes capsid protein p72, which is the most dominant structural component of the virion constituting about 31–33% of the total mass of the virion and is the major antigen detected in ASFV-infected pigs (Bastos et al., 2003; Boshoff et al., 2007; Revilla et al., 2018; Liu Q. et al., 2019). A previous study showed that the *p72* gene nucleotide sequences of MAL/19/Karonga/1–4 showed 100% nucleotide identity with ASFV strains previously described in Tanzania, Zambia, Georgia, China, Vietnam, Estonia, Moldova, Czechia, Belgium, and Poland, which suggests that the *p72* gene is relatively conserved (Hakizimana et al., 2020). The primers used for ASFV detection were designed based on the *p72* gene.

At present, no efficient vaccine or treatment against ASF is available, even though much time, energy, and money have been invested in many countries. Meanwhile, mutant strains of ASFV have been emerging through mutations and recombinations in the genome in order to survive in different environments during the ASFV epidemic process, which hinder the early diagnosis of ASF (Sun et al., 2021; Zhang et al., 2021). Infected pigs have developed atypical clinical symptoms, such as reduced mortality, highly concealed with the long-term prevalence and the spread of ASF in China, which bring challenges to its prevention and control. Therefore, reliable early diagnosis technology and epidemiological monitoring are essential for preventing, controlling, and purifying ASF.

This study constructed a real-time recombinase-aid amplification (RAA) assay for the rapid, on-site, and primary detection of ASFV genomic DNA. Thirty samples were detected, including 12 artificially spiked samples of ASFV, 2 samples of foot-and-mouth disease virus (FMDV), 1 sample of classical swine fever virus (CSFV), 1 sample of porcine reproductive and respiratory syndrome virus (PRRSV), and 14 ASFV-free samples, which were confirmed by the real-time quantitative PCR (qPCR) previously reported (Gallina et al., 2006). The results showed that the sensitivity of the real-time RAA assay was at 10^3^ copies per reaction at 95% probability in 9 min at 39°C. The method was universally specific for three strains of ASFV, and there was no cross-reaction with other pathogens, including FMDV, CSFV, PRRSV, porcine circovirus 2 (PCV2), pseudorabies virus (PRV), and porcine parvovirus (PPV). The coefficient of variation (CV) of repetitive experiments was 0%, and the coincidence rate was 100% compared to real-time qPCR. The real-time RAA assay was then evaluated on 123 field samples compared to the real-time qPCR assay. Fifty-one samples were determined to be positive by the real-time RAA assay, and 52 samples were positive by the real-time qPCR assay (CT values ranging from 20 to 30). The clinical coincidence rate of the real-time RAA assay was 98% compared to the real-time qPCR assay.

Our results demonstrated that the real-time RAA assay, equipped with portable instruments, provided a specific and sensitive platform for the rapid and reliable detection of the ASFV genome. The advantages of this method included the following: the extraction of DNA can be performed in a short time on-site, the template is directly used, a small battery-powered instrument is easily available, and the on-site detection process is finished within an hour. All these suggest that this approach could be used to detect different genotypes of ASFV and play a vital role in the surveillance and the control of ASF.

## Materials and Methods

### Sample Preparation

In this study, 30 samples were used, as described below: the *p72* gene of three strains of ASFV [E70 strain (Spanish), Anhui XCGQ strain, and Georgia 2007/1 strain] was synthesized by the Wuhan GeneCreate Biological Engineering Co., Ltd., and then mixed with blood (*n* = 1), serum (*n* = 2), and lymphatic nodes (*n* = 1), respectively, and 12 artificially spiked samples. The other samples were preserved in our laboratory: FMDV (FMDV/O, FMDV/A), CSFV, and PRRSV, one sample of each virus. Fourteen ASFV-free samples of the liver (*n* = 1), muscles (*n* = 2), lymphatic tissues (*n* = 5), serum (*n* = 3), and blood (*n* = 3) were collected from healthy swine, and these samples were confirmed using the real-time qPCR previously reported (Gallina et al., 2006). One hundred twenty-three field samples were preserved in our laboratory, including blood (*n* = 15), heart (*n* = 10), liver (*n* = 13), spleen (*n* = 9), lung (*n* = 18), kidney (*n* = 14), inguinal lymph nodes (*n* = 13), mesenteric lymph nodes (*n* = 14), and hepatic and gastric lymph nodes (*n* = 17).

### DNA Extraction

For liquid samples, 40 μl blood or serum was transferred into a 1.5-ml centrifuge tube and added with 100 μl lysis buffer B1, mixed fully, and then added with 200 μl lysis buffer B2. For tissue samples, 5–10 mg tissues was lysed with 100 μl lysis buffer B1, manually ground into pieces with a tissue grinder, then added with 200 μl lysis buffer B2, and vortexed to blend completely. Supernatant of 100–150 μl was transferred into another clean centrifuge tube after centrifuging at 12,000 rpm for 2 min and stored at −80°C until use.

### Real-Time Recombinase-Aid Amplification Primers and Probes

Real-time RAA-specific primers and probes were designed according to the *p72* gene sequence of the ASFV-SY18 strain (GenBank: MH713612.1) separated in 2018 in China. Meanwhile, the *p72* gene sequence of the ASFV-SY18 strain was aligned with homologous sequences of the other ASFV strains (GenBank: LR536725.1, MK333181.1, MK333180.1, MH910496.1, MH910495.1, MH713612.1, MK128995.1, MH681419.1, LS478113.1, KJ195685.1, FR682468.1, AY578707.1, AY578702.1, KX354450.1, KM102979.1, KP055815.1, , AM712240.1, EF121428.1, L76727.1, and EF121429.1) to determine the conserved regions of the *p72* gene. Primers and probes were designed aiming to detect different genotypes of ASFV. All primers and probes were synthesized by Sangon Biotech (Shanghai, China). The probes were synthesized with an inverse arrangement of fluorophore [6-carboxyfluorescein (FAM)], quencher [black hole quencher 1 (BHQ-1)], spacer [tetrahydrofuran spacer (THF)], and block elongation [phosphate(P)]. Information on the primers and probes is listed in Table 1.

**TABLE 1:** Primers and probes used for the real-time recombinase-aid amplification (RAA) assay in this study.

Primer names	Sequences (5′–3′)
ASFV RAA Fe1	AATGGATACTGAGGGAATAGCAAGGTTCACGTTCT
ASFV RAA Fe2	GGATACTGAGGGAATAGCAAGGTTCACGTTCTCG
ASFV RAA Fe3	TCTCGTTAAACCAAAAGCGCAGCTTAATCCAGAGC
ASFV RAA Re1	AAATTTCTTTCACAACATTTTCCCGAGAACTCTCA
ASFV RAA Re2	GCCATACCAACCCGAAATTTCTTTCACAACATTTT
ASFV RAA Re3	CGTGCAGCCATACCAACCCGAAATTTCTTTCACAA
ASFV RAA Pe	AGTATTTAGGGGTTTGAGGTCCATTACAGC(FAM-dT) (THF)(BHQ1-dT) AATGAACATTACGTC-P

*RAA Fe and Re, real-time RAA primers; RAA Pe, real-time RAA probe; BHQ1-dT, thymidine nucleotide carrying Black Hole Quencher 1; THF, tetrahydrofuran spacer; FAM-dT, thymidine nucleotide carrying fluorescein; P, block elongation.*

### Generation of pUC57-p72 Standard Plasmid

The ASFV *p72* gene segments (887 bp) were synthesized by Sangon Biotech (Shanghai, China) based on the ASFV genomic sequence (GenBank: MH713612.1) and were inserted into a pUC57 vector to generate standard plasmid, designated as pASFV/RAA. The pASFV/RAA plasmid was extracted using the DNeasy Blood & Tissue Kit (50) (Qiagen, Germantown, MD, United States), and the DNA concentration was measured by Nanovue (GE Life Sciences, Chicago, IL, United States). The DNA copy number were calculated using the following equation (Xia et al., 2014): DNA copy number = (*M* × 6.02 × 10^23^ × 10^–9^)/(*n* × 660). The DNA standard was then aliquoted and stored at −80°C until use.

### Real-Time Recombinase-Aid Amplification Assay

The RAA reactions were performed in a 50-μl volume, including a centrifuge tube of RAA enzyme dry powder, reaction A of RAA 45.5 μl, DNA template 2 μl, and reaction B of RAA 2.5 μl. The detailed protocol is as follows: 45.5 μl RAA reaction A was transferred into the tube with RAA enzyme dry powder and overturned to mix thoroughly. Of the DNA template, 2 μl was added into a centrifuge tube, and then 2.5 μl RAA reaction B was added, the tube lip covered and inverted upside down five to six times to mix the reaction system thoroughly, and then centrifuged for 5–10 s. The assay was performed on a portable fluorescence quantitative PCR instrument (Suzhou Yarui Biotechnology Co., Ltd., Suzhou, China) for 1 cycle at 39°C for 40 s and 40 cycles at 39°C for 30 s. The reaction was completed within 20 min. The sample was positive if the logarithmic amplification curve revealed a *C*_t_ value ≤35; it was judged as a suspicious sample if 3539 or no value. For interpretation, a positive (+) result is when the ASFV genome DNA was detected in the sample and negative (−) when the ASFV genome DNA was not detected.

### Real-Time qPCR Assay

The primers and probes in the real-time qPCR assay were based on a previous report: forward primer, 5′-CCT CGGCGAGCGCTTTATCAC-3′; reverse primer, 5′-GGAAACT CATTCACCAAATCCTT-3′; probe, 5′-CGATGCAAGCTTTAT-3′ (Zsak et al., 2005). All primers and probes were synthesized by Sangon Biotech (Shanghai, China). The assay was performed in a LightCycler 480 II real-time fluorescent quantitative PCR instrument (Roche, Indianapolis, IN, United States) according to the a previously described method (Gallina et al., 2006). The reactions were conducted according to the manufacturer’s instructions in the one-step PrimeScript RT-PCR Kit (TaKaRa, Shiga, Japan). The total reaction in a 20-μl reaction volume contained 10 μl of 2 × one-step RT-PCR buffer III, 0.4 μl of TaKaRa Ex Taq HS, 0.4 μl of PrimeScript RT Enzyme Mix II, 0.4 μl of PCR forward primer, 0.4 μl of PCR reverse primer, 0.8 μl of probes, 2 μl of DNA template, and 5.6 μl RNase-free ddH_2_O. Cycling proceeded at 42°C for 5 min and 95°C for 10 s, followed by 40 cycles of 95°C for 5 s and 60°C for 20 s. A melting curve analysis was performed using specific melting temperatures to verify the uniqueness of the amplified product. The data were analyzed using the LC480 System software.

### Statistical Analysis

Data were provided as the mean and standard deviation (SD). Statistical analysis was performed using PRISM 8.0.2 software (GraphPad Software, San Diego, CA, United States).

## Results

### Sensitivity and Specificity of the Real-Time Recombinase-Aid Amplification Assay

**FIGURE 1:**
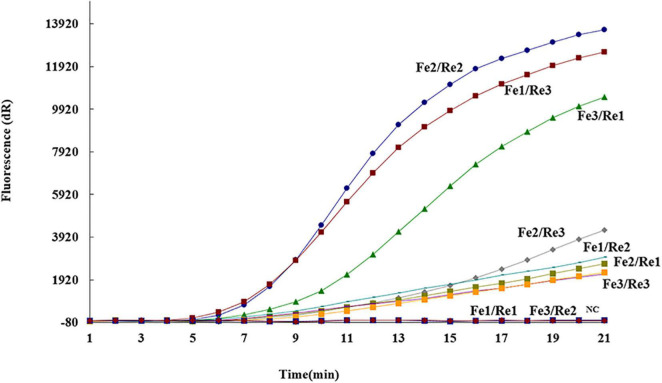
Optimal primer and probe combinations for the real-time recombinase-aid amplification (RAA) assay. Three forward primers (ASFV Fe1–Fe3), three reverse primers (ASFV Re1–Re3), and one probe (ASFV Pe) were used to select the best combination. *NC*, negative control.

To determine the most efficient primer pair for the real-time RAA assay, three forward primers (ASFV Fe1–Fe3) and reverse primers (ASFV Re1–Re3) based on the *p72* gene of ASFV were selected (Table 1). Nine different combinations of the primers (i.e., Fe1/Re1, Fe1/Re2, Fe1/Re3, Fe2/Re1, Fe2/Re2 Fe2/Re3, Fe3/Re1, Fe3/Re2, and Fe3/Re3) were detected with the ASFV Pe probe on 10^4^ genome copies of standard DNA. The results showed that the primer pair ASFV Fe2/Re2 yielded the highest amplification efficiency (Figure 1). Therefore, this primer pair was employed to validate the real-time RAA assay.

**FIGURE 2:**
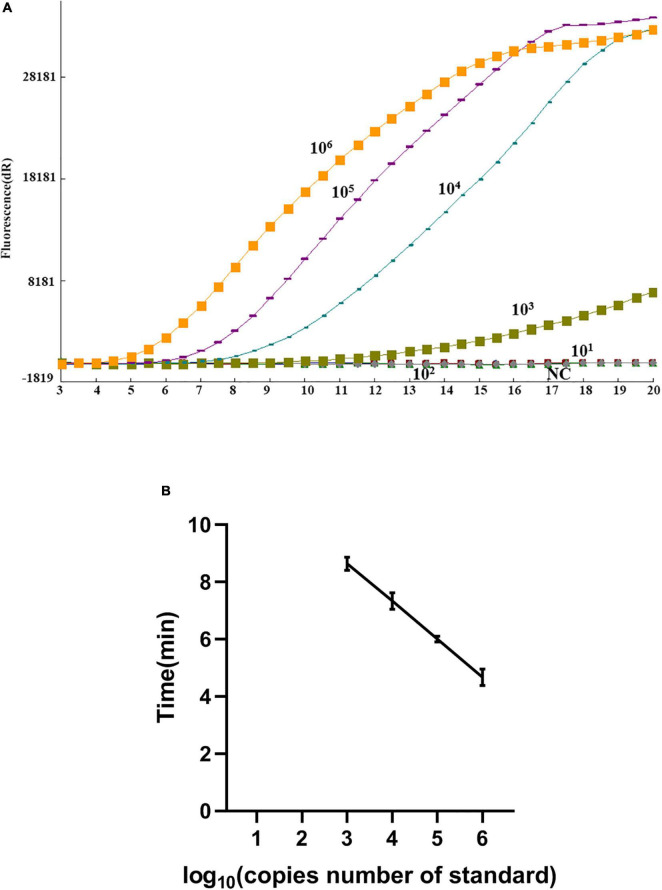
Sensitivity of the real-time recombinase-aid amplification (RAA) assay. **(A)** Amplification curve of the real-time RAA assay over time using a dilution range of 10^6^–10^1^ copies/reaction of pASFV/RAA. **(B)** Reproducibility of the real-time RAA assay. The threshold time is represented as the mean ± standard deviation (SD). The standard regression line was generated based on three datasets. *NC*, negative control.

Serial dilution of the pASFV/RAA plasmid was performed, ranging from 10^1^–10^6^ genome copies per reaction, and then tested using the real-time RAA assay to determine the detection limit. Every run was repeated eight times. The threshold time was plotted against log (detected molecules), and a semi-log regression was calculated using PRISM 8.0.2 software (GraphPad Software). As shown in Figure 2, the dynamic detection range of the assay spans six logs ranging from 6 to 1 log copy per reaction, with the corresponding threshold time ranging from 5 min at 10^6^ copies per reaction to 9 min at 10^3^ copies per reaction. These results indicate that the real-time RAA assay has a wide dynamic range for detecting ASFV genome DNA (Figures 2A,B).

To further evaluate the specificity of the real-time RAA assay, ASFV was evaluated among other detections of swine disease pathogens, including CSFV, PCV2, PRRSV, PRV, PPV, and FMDV. The results showed that no cross-reaction was observed with other viruses, and ASFV strains could be detected by the developed real-time RAA assay (Table 2).

**TABLE 2:** Evaluation of the specificity of the real-time recombinase-aid amplification (RAA) assay.

Virus family	Virus species	Virus strains	Real-time RAA	Real-time qPCR
Asfarviridae	ASFV	SY18	6 min	22 (*C*_t_ value)
Flaviviridae	CSFV	C-strain	−	−
Circoviridae	PCV2	NX strain	−	−
Arteriviridae	PRRSV	CH-1R	−	−
Herpesviridae	PRV	Fa	−	−
Parvoviridae	PPV	AV30	−	−
Picornaviridae	FMDV	FMDV/O/CHA	−	−

*“−” denotes negative result. ASFV, African swine fever virus; CSFV, classical swine fever virus; PCV2, porcine circovirus type 2; PRRSV, porcine reproductive and respiratory syndrome virus; PRV, pseudorabies virus; PPV, porcine parvovirus; FMDV, foot-and-mouth disease virus.*

### Repeatability and Coincidence Experiments of the Real-Time Recombinase-Aid Amplification Assay

Thirty samples were collected to test the performance of the real-time RAA assay, including 12 artificially spiked-samples of ASFV, 2 FMDV samples (a FMDV/O cellular sample and a FMDV/A lymphatic sample), one CSFV sample, one PRRSV sample and 14 ASFV-free samples. Thirty samples were detected by the real-time RAA assay with three repetitions. The results showed that the CV of repetitive experiments was 0% in the intra- and inter-assays, suggesting that the repeatability was good (Tables 3, 4). Meanwhile, 30 samples were used to perform coincidence testing by the real-time RAA assay. The results showed that the coincidence rate was 100% compared to real-time qPCR (Table 5). Taken together, the real-time RAA assay was specific for detecting the ASFV genome, and the sensitivity reached 10^3^ copies per reaction. The CV of repetitive experiments was 0% in the inter- and intra-assays, and the coincidence rate of the real-time RAA assay was 100% compared to the real-time qPCR assay, suggesting that this approach could be used to detect different genotypes of ASFV and play a vital role in its control.

**TABLE 3:** Results of repetitive experiments with the real-time recombinase-aid amplification (RAA) assay.

Sample ID	Viral strains	Real-time RAA		
		Test 1	Test 2	Test 3
S1	ASFV/E70	+ /+/+	+/+/ +	+ /+/+
S2	ASFV/E70	+/+/+	+/+/+	+/+/+
S3	ASFV/E70	+ /+/+	+/+/ +	+ /+/+
S4	ASFV/E70	+/+/+	+/+/+	+/+/+
S5	ASFV/XCGQ	+ /+/+	+/+/ +	+ /+/+
S6	ASFV/XCGQ	+/+/+	+/+/+	+/+/+
S7	ASFV/XCGQ	+ /+/+	+/+/ +	+ /+/+
S8	ASFV/XCGQ	+/+/+	+/+/+	+/+/+
S9	ASFV/2007/1	+ /+/+	+/+/ +	+ /+/+
S10	ASFV/2007/1	+ /+/+	+/+/ +	+ /+/+
S11	ASFV/2007/1	+ /+/+	+/+/ +	+ /+/+
S12	ASFV/2007/1	+ /+/+	+/+/ +	+ /+/+
S13	FMDV/O	−/−/−	−/−/−	−/−/−
S14	FMDV/A	−/−/−	−/−/−	−/−/−
S15	CSFV	−/−/−	−/−/−	−/−/−
S16	PRRSV	−/−/−	−/−/−	−/−/−
S17	ASFV-free	−/−/−	−/−/−	−/−/−
S18	ASFV-free	−/−/−	−/−/−	−/−/−
S19	ASFV-free	−/−/−	−/−/−	−/−/−
S20	ASFV-free	−/−/−	−/−/−	−/−/−
S21	ASFV-free	−/−/−	−/−/−	−/−/−
S22	ASFV-free	−/−/−	−/−/−	−/−/−
S23	ASFV-free	−/−/−	−/−/−	−/−/−
S24	ASFV-free	−/−/−	−/−/−	−/−/−
S25	ASFV-free	−/−/−	−/−/−	−/−/−
S26	ASFV-free	−/−/−	−/−/−	−/−/−
S27	ASFV-free	−/−/−	−/−/−	−/−/−
S28	ASFV-free	−/−/−	−/−/−	−/−/−
S29	ASFV-free	−/−/−	−/−/−	−/−/−
S30	ASFV-free	−/−/−	−/−/−	−/−/−

*“ + “ and “−” denote positive and negative results, respectively. ASFV, African swine fever virus; FMDV, foot-and-mouth disease virus; CSFV, classical swine fever virus; PRRSV, porcine reproductive and respiratory syndrome virus.*

**TABLE 4:** Statistical results of repeatability with the real-time recombinase-aid amplification (RAA) assay.

	Test 1	Test 2	Test 3
No. of repetitions	3	3	3
No. of samples	30	30	30
Within-run CV (%)	0	0	0
Between-run CV (%)	0	0	0

*Coefficient of variation (CV) = (positive samples/total samples) × 100%.*

**TABLE 5:** Coincidence rate between the real-time recombinase-aid amplification (RAA) assay and real-time quantitative PCR (qPCR).

Sample ID	Viral strains	Real-time qPCR	Real-time RAA
S1	ASFV/E70	+	+
S2	ASFV/E70	+	+
S3	ASFV/E70	+	+
S4	ASFV/E70	+	+
S5	ASFV/XCGQ	+	+
S6	ASFV/XCGQ	+	+
S7	ASFV/XCGQ	+	+
S8	ASFV/XCGQ	+	+
S9	ASFV/2007/1	+	+
S10	ASFV/2007/1	+	+
S11	ASFV/2007/1	+	+
S12	ASFV/2007/1	+	+
S13	FMDV/O	−	−
S14	FMDV/A	−	−
S15	CSFV	−	−
S16	PRRSV	−	−
S17	ASFV-free	−	−
S18	ASFV-free	−	−
S19	ASFV-free	−	−
S20	ASFV-free	−	−
S21	ASFV-free	−	−
S22	ASFV-free	−	−
S23	ASFV-free	−	−
S24	ASFV-free	−	−
S25	ASFV-free	−	−
S26	ASFV-free	−	−
S27	ASFV-free	−	−
S28	ASFV-free	−	−
S29	ASFV-free	−	−
S30	ASFV-free	−	−

*“+” and “−” represent positive and negative results, respectively. ASFV, African swine fever virus; FMDV, foot-and-mouth disease virus; CSFV, classical swine fever virus; PRRSV, porcine reproductive and respiratory syndrome virus.*

### Evaluation of the Real-Time Recombinase-Aid Amplification Assay With Clinical Samples

To explore the clinical performance of the real-time RAA assay, 123 field samples preserved in our laboratory were used, including blood, heart, liver, spleen, lung, kidney, inguinal lymph nodes, mesenteric lymph nodes, and hepatic and gastric lymph nodes. The performance of the real-time RAA assay was compared to that of the real-time qPCR assay. Fifty-one samples were determined to be positive by the real-time RAA assay, while 52 samples were positive by the real-time qPCR assay (CT values ranging from 20 to 30). The clinical coincidence rate of the real-time RAA assay was 98% compared to the real-time qPCR assay (Table 6).

**TABLE 6:** Comparison of the real-time recombinase-aid amplification (RAA) assay with the real-time quantitative PCR (qPCR) assay on clinical samples.

Clinical samples	Real-time RAA assay		Real-time qPCR assay	
	Positive	Negative	Positive	Negative
Blood	2	13	2	13
Heart	7	3	7	3
Liver	9	4	9	4
Spleen	8	1	8	1
Lung	7	11	8	10
Kidney	6	8	6	8
Inguinal lymph nodes	7	6	7	6
Mesenteric lymph nodes	4	10	4	10
Hepatic and gastric lymph nodes	1	16	1	16
Total	51	72	52	71

## Discussion

African swine fever is a deadly infectious disease that leads to an acute hemorrhagic fever in domestic and wild pigs, with a mortality rate of up to 100%. Presently, it poses a major concern for the global swine industry due to *trans-*continent transmission of ASF from Africa to Europe and Asia, particularly its introduction and dramatic spread in 2018 in China, which has half of the world’s pig population (Dixon et al., 2019). At present, the characteristics of attenuated strain infection have been emerging, such as the epidemic trend slowing down, low mortality of infection in pigs, and the clinical symptoms not being obvious. ASFV variant strains have been found in different regions in China since 2020. The genome sequences of isolated strains vary, including nucleotide mutations, insertions, deletions, or substitutions. Among them, the mutation frequency of the *CD2v* gene was higher than in other genes, leading to the loss of the red blood cell adsorption characteristics of some strains, weakened virulence, and minor clinical symptoms in infected animals, which bring challenges to ASF prevention and control (Gao et al., 2018; Song et al., 2021). Hence, the development of a rapid diagnostic and accurate detection technology for ASFV is urgent.

Most diagnostic methods based on qPCR require a long sample preparation time, analysis, and expensive instrument, which are not suitable for rapid detection on-site. Serological detection techniques, such as ELISA antibody testing, are suitable for population testing. However, the production of antibodies is delayed. It is difficult to screen pigs through antibody testing in the incubation period. Meanwhile, there are certain deficiencies in the sensitivity and accuracy. Hence, it is essential to develop an early detection and diagnostic method that is sensitive, specific, and suitable on-site with the persistent spread of ASF. This study developed a real-time RAA assay to detect ASFV genomic DNA. The results of 12 artificially spiked samples of ASFV showed that the sensitivity was 10^3^ per reaction, the specificity was 100% compared to real-time qPCR, and no cross-reaction with other pathogens. For the 123 field samples evaluated using the clinical coincidence rate of the real-time RAA assay was 98% compared to the real-time qPCR assay. The entire process was completed within an hour, coupled with a commercial lysis buffer for DNA extraction and a portable detection device suitable for early detection and diagnosis on-site. It has been shown that, among the developed simple and rapid detection methods for ASFV, the loop-mediated isothermal amplification (LAMP) assay can determine at least 330 copies in 75 min (James et al., 2010), polymerase cross-linking spiral reaction (PCLSR) can reach up to 720 copies/μl within 45 min (Wozniakowski et al., 2017), and cross-priming amplification (CPA) can detect 200 copies within 60 min (Gao et al., 2018). The chimeric DNA/LNA-based biosensor processed up to 40 samples (one sample at a time, per analysis of about 5 min), and the limit of detection was 178 copies/μl (Biagetti et al., 2018). The pen-side molecular diagnostic UPL (Universal ProbeLibrary) assay based on the qPCR technique required at least 35 min (Liu L. et al., 2019). CRISPR-Cas12a for ASFV detection was developed, and the sensitivity was about 10 times higher (1.16 copies/μl) than that of the commercial quantitative PCR kit or the OIE-recommended qPCR, and the entire process was finished within 2 h. However, this method has a certain limit for on-site detection (Wang et al., 2020). The sensitivity of the time-resolved fluorescence immunoassay (TRFIA) for ASFV antigen detection was 0.015 ng/ml. The protocol time was only 45 min, which is suitable for the laboratory diagnosis of ASFV infection (Chen et al., 2021). Together, the above-mentioned methods are time-consuming and unsuitable for rapid detection on-site.

However, the recombinase-based detection method shortens the reaction time from 45–120 min for LAMP, PCLSR, CPA, and PCR to 10–30 min and maintains the detection limit of qPCR. Recombinase polymerase amplification (RPA) is an isothermal nucleic acid amplification technology used to detect nucleic acid instead of PCR without an expensive thermocycler, which has been successfully applied for the detection of pathogens, such as Ebola virus, FMDV, avian influenza virus (AIV), and *Brucella* (Zhai et al., 2020). Similarly, an RAA assay combined with a portable instrument detected as few as 10 copies per reaction within 20 min, and DNA extraction was performed *via* a commercial kit (Wang et al., 2021). A directly visualized recombinase polymerase amplification–SYBR Green I method was developed with a detection limit of 10^3^ copies/μl of ASFV in 15 min at 35°C, which can be used as a robust tool for the detection of ASFV genomic DNA (Zhang et al., 2020). PRA combined with lateral flow dipstick (LFD) was developed. This assay was accomplished within 10 min at 36–44°C, and the limit of detection was 10^2^ copies per reaction, providing an efficient method for ASFV detection (Zhai et al., 2020). The combination of RPA and RAA yielded 93.4 and 53.6 copies per reaction within 16 min, respectively. However, DNA extraction was performed using an instrument, and the samples required pretreatment (Fan et al., 2020).

In recent years, the RAA/RPA method has been applied for the detection of many pathogens, such as *Vibrio harveyi*, PCV2, enterovirus 71 (EV71), and coxsackievirus A16 (CA16) (Wang et al., 2017; Li et al., 2019; Pang et al., 2019). More and more detection and diagnostic methods for ASFV are emerging with the persistent infection of ASFV and the non-classical symptoms. RAA/RPA seems to have more potential as an early diagnostic method for ASFV because its limit detection is the same as that of the OIE-recommend qPCR. The entire process is completed on-site within a short time *via* a portable testing device. Hence, it still requires extensive validation and optimization to provide a rapid and reliable strategy for early detection, such as for disposal, movement control, disinfection, and contamination to the environment.

Altogether, our data demonstrated that the real-time RAA, coupled with a portable detection device and a commercial lysis buffer for DNA extraction, can be a sensitive, specific, rapid, and reliable tool for ASFV genomic DNA detection on-site, promoting the control and surveillance of ASF in the future.

## Data Availability Statement

The raw data supporting the conclusions of this article will be made available by the authors, without undue reservation.

## Author Contributions

YY, ZDZ, YML, YJL, and YSW designed the experiments and wrote and revised the manuscript. YSW performed the experiments. YY and XQ analyzed the data. YR, YSW, ML, ZXZ, and RZ prepared and performed experiments on the clinical samples. All authors contributed to the article and approved the submitted version.

## Conflict of Interest

The authors declare that the research was conducted in the absence of any commercial or financial relationships that could be construed as a potential conflict of interest.

## Publisher’s Note

All claims expressed in this article are solely those of the authors and do not necessarily represent those of their affiliated organizations, or those of the publisher, the editors and the reviewers. Any product that may be evaluated in this article, or claim that may be made by its manufacturer, is not guaranteed or endorsed by the publisher.
